# 

**Published:** 1899-11-18

**Authors:** 


### NOTICE TO CORRESPONDENTS.

All MS., Letters, Books for Review, and other matters intended for the Editor should be addressed The Editor, "The Hospital," The Hospital Building, 28 & 29, Southampton Street, Strand, W.C.

The Editor cannot undertake to return rejected MS., even when accompanied by stamped directed envelope.

**Notice to Advertisers and Subscribers.**

All Advertisements, Orders for Copies of The Hospital, and Business Communications should be addressed to **THE MANAGER (Not to the Editor)**, The Hospital, The Hospital Building, 28 & 29, Southampton Street, Strand, London, W.C.

The Cost of Subscription, post free, is as follows:—Per week, 2½d.; per quarter, 2s. 8d.; per half-year, 5s. 3d.; per annum, 10s. 6d. Foreign:—Per annum, 15s. 2d., payable, in all cases, in advance.

**NOTICE.—Vol. XXVI. of "The Hospital" (April, 1899, to September, 1899), in handsome cloth binding, is now on sale at the Office of this Journal, price 7s. 6d. Cases for binding the Numbers contained in this Volume 1s. 6d. Index to Vol. XXVI., 2½d., post free.**

**The Monthly Part for December will be ready on December 1st. Price 6d., by post 8d.**

### NOTES ON BOOKS.

Dr. Arthur Larking has published a little book entitled "Notes on Folkestone" (London: J. and A. Churchill), which serves as a handy guide to that town and neighbourhood. It contains much useful information on questions of temperature, rainfall, sunshine, and the other matters which go to make up the attractiveness of a health resort, and gives a variety of hints on selecting apartments and on the class of cases which are found most suitable for treatment at Folkestone. In regard to the remark so often made that "The air of Folkestone is so strong," he says that one is puzzled to define what is meant by "strong air." But, at any rate, one effect of such air is, as everyone knows, to produce a feeling of drowsiness and a tendency to sleep. In regard to this Dr. Larking very properly says, "Persons who are naturally indolent and fond of taking things easy are apt to give way to this feeling of drowsiness, and they dose about the hotel or board house and neglect the proper amount of exercise. They then get bilious, especially if their appetite is good, and say Folkestone does not suit them!" We fancy this applies to many a health resort besides Folkestone.

The Maintenance of Health in Egypt, by Dr. Bentley (Simpkin, Marshall, Hamilton, Kent, and Co.), is a little shilling volume which, although somewhat of the nature of an advertisement of an hotel at Helouan, is still a very good little book. It is full of information about Egypt, and of hints on the subject which forms its title; it is well illustrated, and will be found very interesting by intending visitors.

### BOOKS RECEIVED.

Germer Baillière et Cie.

"Dictionnaire de Physiologie." Par Charles Richet.

Baillière, Tindall, and Cox.

"An Introduction to Diseases of the Nervous System." By H. Campbell Thomson, M.D.Lond., M.R.C.P.

"A Manual of Surgery for Students and Practitioners." By William Rose, M.B., B.S.Lond., F.R.C.S., and Albert Carless, M.S.Lond., F.R.C.S.

Isbister and Co.

"The Sunday Magazine," 1899. "Good Words," 1899. Edited by the Very Rev. Donald Macleod, D.D.

S. W. Partridge and Co.

"The Zenana," 1898-9.

Society for Promoting Christian Knowledge.

"Matter, Ether, and Motion." By A. E. Dolbear, Ph.D. English Edition. Edited by Professor Alfred Lodge.

### SCRAPS AND GLEANINGS.

Lord Rothschild has given a site at Tring for an isolation hospital which the Urban District Hospital are proposing to erect.

A provisional committee of medical men has been formed for the establishment of a Dublin hospital for diseases of the skin.

The Metropolitan Asylums Board reckons to have to deal with each week from 5,000 to 6,000 cases in the infectious hospitals, which number twelve.

The Grantham Hospital workers have had a very successful year, being able to hand over to the hospital a sum of £200 as the result of their efforts.

The Central Red Cross Committee have accepted a gift of two cases (about 3,000 rations) Maggi's Consommé, from Messrs. Cosenza and Co., for the use of the sick and wounded in the South African campaign.

The new infectious hospital at Baghill is now almost completed, and if any cases of small-pox were to arise in the district under the jurisdiction of the Pontefract Board of Guardians they could be accommodated at this new hospital.

A considerable sum of money has been left by the late Mr. Godfrey Ermen to local Manchester educational and charitable institutions. At present the amount at their disposal has not been ascertained, but a bequest of £1,000 has been made to the Eccles and Patricroft Hospital.

The total cost of proposed alterations and extensions at the Stockton and Thornaby Hospital is £7,000. Of this sum £1,500 is in hand, and £1,250 is promised, which will be increased to £1,500 if the workmen of Stockton and Thornaby subscribe £1,000.

The house-to-house collection last year on behalf of the Worcester Medical Charities and Orphan Asylum amounted to £250, whilst the previous year's collection was only £100. It is hoped that this year's collection will prove still more successful.

The special commissioner of the *Lancet* has severely criticised the Bath Royal Mineral Water Hospital, which it is stated is in a very bad condition in regard to its sanitary arrangements. This institution was first opened to patients in 1742, and now contains 102 beds for males and 70 for females.

At a recent special meeting of the North Ormesby Cottage Hospital the following resolution was unanimously adopted: "That the representatives of the workmen on the House Committee of this hospital invite their fellow workmen to undertake the cost of furnishing and equipping the new Jubilee wing."

The Suffolk General Hospital has found it expedient to revert back to the old regulation which provided that each annual subscriber of £2 2s. should be entitled to obtain admission for one in-patient and two out-patients. Since 1889 two in-patients and two out-patients have been allowed on each £2 2s. annual subscription, but this arrangement has been found in practice to work unsatisfactorily.

At the recent Ledbury Fair Mr. John Studt offered to the committee of the Ledbury Cottage Hospital the use of his electrically-lighted switchback as a benefit for that institution. From six until ten o'clock the machine was in constant motion, and the result of Mr. Studt's kind offer was the handsome sum of £18 16s. 3d. for the hospital funds. Mr. Studt had in the previous week given similar assistance to the hospital at Tewkesbury, where £14 9s. was realised.

### THE STUDENT OF MEDICINE.

### APPOINTMENTS.

H. Bankes Price, M.R.C.S., L.R.C.P., appointed Honorary Medical Officer to the Coventry and Warwickshire Hospital. F. F. Barnardo, M.A., M.B., Ch.B., appointed House Surgeon to Royal Maternity and Simpson Memorial Hospital, Edinburgh. N. Close, L.R.C.S.I., L.R.C.P., appointed Medical Officer for the No. 2 District of the Chard Union. J. T. Davies, M.D.Mich., M.R.C.S., L.R.C.P., appointed as Clinical Assistant to the Chelsea Hospital for Women. A. D. Fordyce, M.B., B.Ch., appointed House Surgeon to Royal Maternity and Simpson Memorial Hospital, Edinburgh. F. H. Gervis, M.D.Brux., M.R.C.S., L.R.C.P.Lond., appointed Clinical Assistant to Paddington Green Children's Hospital. W. Hall, M.B., B.Ch., appointed Senior Demonstrator in Physiology at the Owens College, Manchester. M. G. McElligott, D.P.H., L.R.C.S.I., appointed Medical Superintendent of Belper Isolation Hospital. H. R. Phillips, M.B., Ch.B.Edin., appointed House Surgeon to the Children's Hospital, Paddington Green, W. T. E. Saxby, L.R.C.P., L.R.C.S.Edin., L.F.P.S.G., appointed Medical Officer to the Parish of Uist, Shetland, N.B. R. Serjeant, M.R.C.S.-Eng., L.R.C.P.Lond., appointed Assistant Medical Officer to the Camberwell House Asylum. W. B. Smith, B.A., M.D., B.Ch., B.A.O.Dub., appointed Deputy Medical Superintendent to the Belper Isolation Hospital. H. L. Willis, M.R.C.S.Eng., L.R.C.P.-Lond., appointed Junior Assistant Medical Officer to the Govan District Asylum.

